# P2Y_12_ receptor blockade synergizes strongly with nitric oxide and prostacyclin to inhibit platelet activation

**DOI:** 10.1111/bcp.12826

**Published:** 2016-02-10

**Authors:** Melissa V. Chan, Rebecca B. M. Knowles, Martina H. Lundberg, Arthur T. Tucker, Nura A. Mohamed, Nicholas S. Kirkby, Paul C. J. Armstrong, Jane A. Mitchell, Timothy D. Warner

**Affiliations:** ^1^ The William Harvey Research Institute, Barts and the London School of Medicine and DentistryQueen Mary University of LondonLondonUK; ^2^ Qatar Foundation Research and Development DivisionDohaQatar; ^3^ National Heart & Lung InstituteImperial College LondonLondonUK

**Keywords:** blood platelets, endothelium, epoprostenol, nitric oxide, purinergic P2Y receptor antagonists

## Abstract

**Aims:**

*In vivo* platelet function is a product of intrinsic platelet reactivity, modifiable by dual antiplatelet therapy (DAPT), and the extrinsic inhibitory endothelial mediators, nitric oxide (NO) and prostacyclin (PGI_2_), that are powerfully potentiated by P2Y_12_ receptor blockade. This implies that for individual patients endothelial mediator production is an important determinant of DAPT effectiveness. Here, we have investigated this idea using platelets taken from healthy volunteers treated with anti‐platelet drugs.

**Methods:**

Three groups of male volunteers (*n* = 8) received either prasugrel (10 mg), aspirin (75 mg) or DAPT (prasugrel + aspirin) once daily for 7 days. Platelet reactivity in the presence of diethylammonium (Z)‐1‐(N,N‐diethylamino)diazen‐1‐ium‐1,2‐diolate (DEA/NONOate) and PGI_2_ was studied before and following treatment.

**Results:**

*Ex vivo*, PGI_2_ and/or DEA/NONOate had little inhibitory effect on TRAP‐6‐induced platelet reactivity in control conditions. However, in the presence of DAPT, combination of DEA/NONOate + PGI_2_ reduced platelet aggregation (74 ± 3% to 19 ± 6%, *P* < 0.05). *In vitro* studies showed even partial (25%) P2Y_12_ receptor blockade produced a significant (67 ± 2% to 39 ± 10%, *P* < 0.05) inhibition when DEA/NONOate + PGI_2_ was present.

**Conclusions:**

We have demonstrated that PGI_2_ and NO synergize with P2Y_12_ receptor antagonists to produce powerful platelet inhibition. Furthermore, even with submaximal P2Y_12_ blockade the presence of PGI_2_ and NO greatly enhances platelet inhibition. Our findings highlight the importance of endothelial mediator *in vivo* modulation of P2Y_12_ inhibition and introduces the concept of refining *ex vivo* platelet function testing by incorporating an assessment of endothelial function to predict thrombotic outcomes better and adjust therapy to prevent adverse outcomes in individual patients.

## What is Already Known About This Subject

- Platelet function is a product of intrinsic platelet reactivity.
- This can be modified by dual anti‐platelet therapy (DAPT), but also by the influence of the endothelial mediators, nitric oxide (NO) and prostacyclin (PGI_2_).
- NO and PGI_2_ also independently amplify each other's effects.

## What this Study Adds

- Three way synergy between PGI_2_, NO and P2Y_12_ receptor antagonism produces powerful platelet inhibition.
- Even with submaximal (25%) P2Y_12_ blockade, the presence of PGI_2_ and NO greatly enhances platelet inhibition.
- Assessing endothelial mediator production and associations to platelet cyclic nucleotides *in vivo* could improve thrombotic outcomes in individual patients.

## Introduction

Compromise in the integrity of the vascular endothelium precipitates rapid platelet activation as platelets become exposed to sub‐endothelial collagen and tissue factor. This activation is driven by a cascade of complex intracellular signalling pathways leading to the production of secondary platelet agonists, notably thromboxane (TX) A_2_ and ADP 1, 2. Dual anti‐platelet therapy (DAPT) is recommended for the secondary prevention of atherothrombotic events in patients with acute coronary syndromes or following percutaneous coronary intervention 3, 4 and targets these two pathways with a P2Y_12_ receptor antagonist, such as clopidogrel, prasugrel or ticagrelor, and aspirin. The P2Y_12_ receptor blockers inhibit platelet aggregation by blocking the amplifying effects of ADP acting on platelet P2Y_12_ receptors 5, while aspirin targets the TXA_2_‐dependent pathway by inhibiting the cyclooxygenase (COX) enzyme within platelets 6. Whilst DAPT is associated with an improvement in patient outcomes, thrombotic events do still occur. An often explored hypothesis is that the risk of experiencing a thrombotic event is associated with the level of platelet blockade, i.e. those individuals with less effective blockade provided by aspirin and, particularly P2Y_12_ receptor blockers, are more at risk of thrombotic events. However, studies have failed to show any benefits from *ex vivo* monitoring of platelet function and subsequent tailoring of treatment in patients receiving dual anti‐platelet therapy 7, 8, 9, 10. This failure is possibly because the *ex vivo* platelet tests used in these trials do not consider the environment in which platelets reside *in vivo*. Namely that within the circulation endothelium‐derived autacoids, nitric oxide (NO) and prostacyclin (PGI_2_), reduce platelet reactivity and prevent inappropriate platelet activation 11, 12. Indeed, within the circulation each platelet is balanced by approximately 50 endothelial cells (e.g. 1.25 trillion platelets *vs.* 60 trillion endothelial cells in a 70 kg man) 13.

NO diffuses freely into platelets activating guanylyl cyclase (GC) to increase intracellular cGMP levels 14, while PGI_2_ binds to IP receptors activating adenylyl cyclase (AC) to increase intracellular cAMP levels 15. Elevations in the intracellular levels of individual cyclic nucleotides promotes a generalized inhibition of platelet function 16 and the two pathways synergize to produce particularly strong inhibition 12. NO and PGI_2_ also individually synergize with P2Y_12_ inhibition producing robust anti‐aggregatory platelet effects 17, 18.

Taking account of the above observations we hypothesized that within the circulation the levels of endothelium‐derived mediators are an important determinant of the efficacy of DAPT. Therefore, for individual patients *in vitro* measures of platelet reactivity do not accurately predict the *in vivo* effectiveness of DAPT due to the confounding of differences in endothelial mediator production. To test this hypothesis we added NO and PGI_2_ to standard *ex vivo* tests of platelet function in blood taken from healthy volunteers receiving anti‐platelet therapies.

## Methods

### Study participants

Twenty‐four healthy, non‐smoking male volunteers (aged 18–40 years) were recruited and participated in the study. Health status was determined though medical history and physical examination, including blood pressure, pulse rate, blood chemistry and urinalysis. Volunteers with normal clinical profiles were included in the study. The study was approved by St Thomas's Hospital Research Ethics Committee (Ref. 07/Q0702/24) and all volunteers gave written consent before entering the study.

### Study protocol

Healthy volunteers abstained from aspirin, non‐steroidal anti‐inflammatory drugs (NSAIDs) and any other anti‐platelet therapy for 14 days before commencing the study. The volunteers were divided into groups of eight. The first group received aspirin (75 mg; Nu‐Seals Cardio 75, Alliance Pharmaceuticals Ltd, Chippenham, UK), the second prasugrel (10 mg; Effient®, Eli Lilly, RA Houten, The Netherlands) and the third both aspirin (75 mg) and prasugrel (10 mg) to represent DAPT for 7 days. Adherence was assessed by interview. Blood samples were collected before and after drug treatment.

### Blood collection

Blood for platelet aggregation was collected by venepuncture into tri‐sodium citrate (0.32% final; Sigma, Poole, Dorset, UK). Platelet‐rich plasma (PRP) was obtained by centrifugation at 175 × *g* for 15 min at 25 °C. Platelet‐poor plasma (PPP) was obtained by centrifugation of PRP at 15 000 × *g* for 2 min. All experiments were completed within 2 h of blood collection.

### Incubation with platelet function inhibitors

For *in vitro* incubation experiments, PRP was treated with either vehicle (0.5% DMSO) or the P2Y_12_ receptor blocker prasugrel active metabolite (PAM; a kind gift of AstraZeneca) at 1.5 μm, 3 μm or 6 μm, to represent 25%, 50% or 100% of the concentration needed for complete P2Y_12_ receptor blockade, respectively, in the absence or presence of aspirin (acetylsalicylic acid, ASA, 30 μm) for 30 min at 37 °C.

### Light transmission aggregometry (LTA)

Baseline aggregation of PRP to arachidonic acid (AA, final concentration, 1 mm, Sigma), adenosine diphosphate (ADP, 5–20 μm, Labmedics, Salford, Manchester, UK), Horm collagen (0.4 and 10 μg ml^−1^, Nycomed, Linz, Austria) and U46619 (10 μm, Cayman Chemical Company, Ann Arbor, MI, USA) was measured in a Bio/Data PAP‐8E turbidimetric aggregometer (1200 rev min^−1^, 37 °C; Alpha Laboratories, Eastleigh, UK) before and following treatment. Aggregations to TRAP‐6 amide specific for PAR1 (TRAP‐6, 25 μm, SFLLRN, Bachem, Bubendorf, Switzerland) or Horm collagen (4 μg ml^−1^) after pre‐incubation (1 min, 37 °C) with the NO donor diethylammonium (Z)‐1‐(N,N‐ diethylamino)diazen‐1‐ium‐1,2‐diolate (DEA/NONOate, 100 nm, Sigma) and/or prostacyclin (PGI_2_, 1 nm, R&D systems, Abingdon, UK) or vehicle (NaOH, 10 mm, Sigma) were also recorded. Using an NO measuring system (iNO600, Harvard apparatus) we showed 83 nm NO release at 2 min and 154 nm at 4 min when 100 nm DEA/NONOate was incubated at pH 7.4.

### Isobolographic analysis

Inhibitory concentration curves for PGI_2_ (1–8 nm) or DEA/NONOate (10 nm – 1 μm) against aggregation induced by TRAP‐6 (25 μm) or collagen (30 μg ml^−1^) in the presence of vehicle or PAM (6 μm) were constructed with data fitted to a logistic equation using least squares method (Prism 6.0e, GraphPad Software, La Jolla, CA, USA). Derived data were used to generate isobolograms 18, 19.

### ADP + ATP secretion

PRP was pre‐incubated for 1 min with DEA/NONOate, PGI_2_ or vehicle in an optical lumi‐aggregometer (560 CA, Chronolog, Havertown, PA, USA). ADP + ATP secretion was evaluated by luminescence in the presence of Chrono‐Lume reagent (0.2 μm luciferin/luciferase, Chronolog) after stimulation with TRAP‐6 (25 μm) or collagen (4 μg ml^−1^).

### P‐selectin expression and GPIIb/IIIa activation

PRP, pre‐incubated with PAM or vehicle, as described earlier, was incubated with PGI_2_, DEA/NONOate or vehicle and then activated with TRAP‐6 (25 μm) with gentle mixing at 37 °C. After 2 min, the reaction was stopped by dilution with a 10‐fold excess of cold saline. Platelets were immediately stained with anti‐CD61‐allophycocyanin (CD61‐APC, eBioscience, Hatfield, UK), PAC‐1‐FITC (BD Bioscience, Oxford, UK), and anti–P‐selectin‐PE (eBioscience) for 15 min at 4 °C and then fixed in 2% (v/v) formalin (Sigma). PAC‐1‐FITC and anti‐P‐selectin‐PE immunoreactivity was measured by flow cytometry using a FACSCalibur instrument (Becton Dickinson, Oxford, UK). Representative histograms are shown in Supplementary Figure 5A–D.

### VASP phosphorylation

PAM or vehicle‐treated PRP was stimulated with collagen (4 μg ml^−1^) or TRAP‐6 (25 μm) in the presence of PGI_2_, DEA/NONOate or vehicle. After 4 min, the reaction was stopped with methanol‐free formaldehyde (2% final, Fisher Scientific). Platelets were permeabilized (0.2% Triton X‐100, Sigma) and incubated with anti‐vasodilator stimulated phosphoprotein (VASP)‐P(Ser^239^) primary antibody (Enzo Life‐sciences, Exeter, UK), Alexa647‐conjugated secondary antibody (Invitrogen, Paisley, UK) and FITC‐conjugated anti‐CD42b (eBioscience, Hatfield, UK), for 30 min each, in turn, before the platelet pellet was resuspended in 0.9% saline. VASP‐P(Ser^239^) immunoreactivity was measured by flow cytometry, using a FACS‐Calibur instrument (Becton Dickinson). Representative histograms are shown in Supplementary Figure 5E,F.

### cAMP and cGMP measurements

PAM or vehicle‐treated PRP was stimulated with collagen (4 μg ml^−1^) or TRAP‐6 (25 μm) in the presence of PGI_2_, DEA/NONOate or vehicle. After 4 min, platelets were lyzed with Triton‐X‐100 (0.625%) and treated with iso‐butylmethylxanthine (IBMX, 500 μm) and potassium fluoride (0.5 m). cAMP and cGMP concentrations were determined by homogenous time‐resolved fluorescence‐based competitive immunoassays (Cisbio Bioassays, Codolet, France).

### Statistics and data analysis

Data were analyzed using Prism 6.0e. Summary data (I*C*_50_, E*C*_50_) were obtained by fitting of data to a logistic equation and tested by Student's *t*‐test (two groups) or one way anova (>two groups). Flow data were analyzed using FlowJo v8.7 (Treestar, Ashland, USA) where the ‘single platelet’ population was gated based on forward scatter and CD61‐APC immunoreactivity (FL‐4 mean fluorescence intensity). Statistical significance was determined by two way anova with Dunnett's *post hoc* test unless otherwise stated and data sets considered different if *P* < 0.05.

P2Y_12_ nomenclature conforms to the BJCP guidelines.

## Results

### Light transmission aggregation responses following in vivo mono and dual anti‐platelet therapy

In individuals taking aspirin, standard LTA responses to AA (1 mm) were strongly inhibited, as were responses to collagen. Responses to ADP (5 μm) were also significantly reduced, although to a lesser degree, while those to U46619 (10 μm) were unaffected (Supplementary Figure 1A). In individuals taking prasugrel, aggregatory responses to AA, collagen, ADP and U46619 were all significantly reduced (Supplementary Figure 1B). Aggregations induced by AA, collagen and ADP were abolished in individuals taking DAPT (Supplementary Figure 1C) while responses to U46619 were strongly reduced.

### Platelet aggregation, ATP secretion, P‐selectin expression and GPIIb/IIIa activation in the presence of PGI_2_ and DEA/NONOate together with DAPT

**Figure 1:**
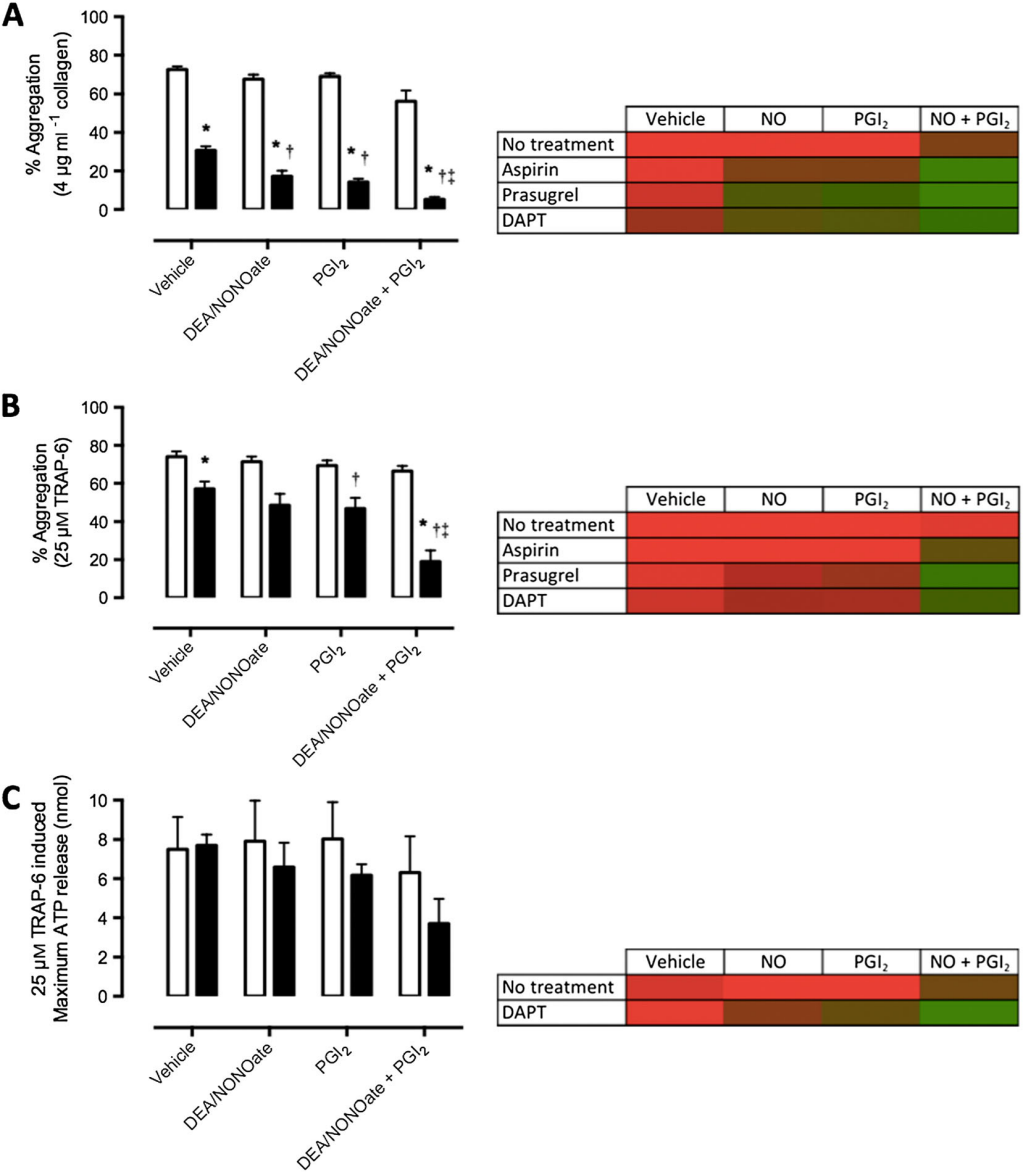
Interactions of NO and PGI_2_ with DAPT: platelet aggregation and ATP release. Bar graphs and heatmaps of platelet aggregation in response to (A) collagen (4 μg ml^−1^), and (B) TRAP‐6 amide (25 μm), and (C) ATP release in response to TRAP‐6 amide (25 μm). Aggregometry was conducted before and after 7 days DAPT (aspirin, 75 mg, plus prasugrel, 10 mg). Aggregometry conducted in the presence of vehicle (NaOH, 10 mm), NO (100 nm), PGI_2_ (1 nm), or NO + PGI_2_. Data are presented as final aggregation (%, mean ± SEM) or ATP release (nm, mean ± SEM). Heatmaps indicate maximum aggregation or ATP release with red and minimum aggregation or ATP release with green. *n* = 8 for all. Significance is shown as * *P* < 0.05 *vs*. non‐treated, † *P* < 0.05 *vs*. NaOH DAPT‐treated ‡ *P* < 0.05 *vs*. PGI_2_ DAPT‐treated. (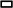) Pre‐treatment , (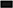) DAPT

In blood from individuals before DAPT, PGI_2_ (1 nm), DEA/NONOate (100 nm) or DEA/NONOate + PGI_2_ had little effect upon platelet aggregation. Following DAPT, collagen (4 μg ml^−1^)‐induced aggregation was significantly reduced from 73 ± 2% to 31 ± 2% (*P* < 0.05, Figure 1A and Supplementary Figure 2A), as seen previously with a lower concentration of collagen (0.4 μg ml^−1^, Supplementary Figure 1A). TRAP‐6 (25 μm)‐induced aggregation was, however, unaffected by DAPT unless DEA/NONOate + PGI_2_ was present, when it was substantially reduced (67 ± 3% to 19 ± 6%, *P* < 0.05, Figure 1B). In parallel experiments, we used lumi‐aggregometry to quantify ADP + ATP release as a measure of dense granule secretion (Supplementary Figure 2B). These studies indicated that TRAP‐6 induced dense granule secretion was unaffected in individuals receiving DAPT administration but was reduced with the further addition of DEA/NONOate + PGI_2_ (6.3 ± 1.9 to 3.7 ± 1.3 nm, *P* < 0.05, Figure 1C). Likewise, this was seen in collagen‐induced ATP release after aspirin or prasugrel alone (Supplementary Figure 4).

Similar effects were found in individuals receiving prasugrel, in whom aggregation in response to TRAP‐6 was significantly reduced (63 ± 3% to 7 ± 3%, *P* < 0.05, Supplementary Figure 3D) by the addition of DEA/NONOate + PGI_2_. Treatment with aspirin alone significantly reduced collagen‐induced aggregation in the presence of both DEA/NONOate (58 ± 9% to 36 ± 8%, *P* < 0.05, Supplementary Figure 3A) and PGI_2_ (66 ± 5% to 35 ± 7, *P* < 0.05, Supplementary Figure 3A). Following treatment with aspirin, TRAP‐6‐induced aggregation was unaffected by DEA/NONOate or PGI_2_, but was reduced by DEA/NONOate + PGI_2_ (58 ± 8% to 28 ± 9%, *P* < 0.05, Supplementary Figure 3B). To aid in visualization of results these data are also expressed as heatmaps (Supplementary Figure 3E, F *)*.

In individuals receiving DAPT, TRAP‐6‐induced P‐selectin expression (geometric mean fluorescence index, MFI, 29.0 ± 7.5 units to 2.4 ± 0.6 units, *P* < 0.05, Supplementary Figure 6A) and PAC‐1 binding (12.4 ± 3.4 units to 1.3 ± 0.5 units, *P* < 0.05, Supplementary Figure 6B) were significantly reduced. This pattern was similar with regard to P‐selectin expression (25.0 ± 6.1 units to 8.8 ± 3.4 units, *P* < 0.05) and PAC‐1 binding (19.2 ± 3.7 units to 1.6 ± 0.3 units, *P* < 0.05) in individuals receiving prasugrel only. Treatment with aspirin alone had no effect on either P‐selectin or PAC‐1 binding.

### Effects of PGI_2_ and DEA/NONOate on platelet aggregation and ATP release in the presence of submaximal P2Y_12_ antagonism is sufficient to produce platelet inhibition

PRP was taken from healthy volunteers and pre‐treated *in vitro* with prasugrel‐active metabolite (PAM, 1.5 μm, 3 μm and 6 μm) to represent 25%, 50% and 100% of the concentration of PAM required for total P2Y_12_ receptor inhibition in the absence (Table 1A) and presence (Table 1B) of aspirin. Aggregation in response to ADP (20 μm) was increasingly inhibited with increasing levels of PAM: control, 73 ± 5%; aspirin, 51 ± 7%; aspirin + PAM‐25%, 33 ± 11%; aspirin + PAM‐50%, 23 ± 8%; aspirin + PAM‐100%, 7 ± 1% (*P* < 0.05).

**Table 1A:** *In vitro* effects of aspirin and PAM on platelet aggregation. PRP from healthy volunteers (*n* = 4) was treated with PAM (1.5 μm, 3 μm and 6 μm) to represent 25%, 50% and 100% maximum concentration for total P2Y_12_ receptor inhibition, respectively. Tables show % final aggregation in response to ADP (20 μm), collagen (4 μg ml^−1^) and TRAP‐6 (25 μm) in the presence of vehicle (NaOH, 10 mm), NO (100 nm), PGI_2_ (1 nm) or NO + PGI_2_ in the (A) absence and (B) presence of aspirin (30 μm).

	**% of maximum concentration for total P2Y**_**12**_ **receptor inhibition**	**Vehicle**	**NO**	**PGI**_**2**_	**NO + PGI**_**2**_
**ADP (20 μm** **)**	0%	80 ± 6	75 ± 2	72 ± 5	23 ± 6 *, †
	25%	70 ± 5	49 ± 13	30 ± 7 *	12 ± 2 *
	50%	45 ± 15	8 ± 3 *	4 ± 1 *	0 ± 0 *
	100%	4 ± 1	0 ± 0 *	0 ± 0 *	0 ± 0 *
**Collagen (4 μg ml** ^**−1**^ **)**	0%	79 ± 4	74 ± 4	74 ± 4	50 ± 10 *, †
	25%	74 ± 3	60 ± 10	58 ± 6	21 ± 10 *, †
	50%	71 ± 4	43 ± 14 *	36 ± 8 *	6 ± 2 *, †
	100%	56 ± 11	23 ± 9 *	22 ± 6 *	4 ± 1 *
**TRAP‐6 (25 μm)**	0%	69 ± 2	70 ± 2	67 ± 2	63 ± 3
	25%	67 ± 2	63 ± 3	61 ± 2	39 ± 10 *, †
	50%	65 ± 4	63 ± 0	55 ± 3	20 ± 6 *, †
	100%	59 ± 3	36 ± 6 *	35 ± 3 *	4 ± 2 *, †

**Table 1B:** 

	**% of maximum concentration for total P2Y**_**12**_ **receptor inhibition**	**Vehicle**	**NO**	**PGI**_**2**_	**NO + PGI**_**2**_
**ADP (20 μm)**	0%	73 ± 5	59 ± 5	52 ± 10	26 ± 13 *
	aspirin +0%	51 ± 7	37 ± 9 *	38 ± 10 *	13 ± 9 *, †
	aspirin +25%	33 ± 11	16 ± 8	17 ± 7	5 ± 4 *
	aspirin +50%	23 ± 8	6 ± 4	9 ± 4	1 ± 1
	aspirin +100%	7 ± 1	0 ± 0 *	0 ± 0 *	0 ± 0 *
**Collagen (4 μg** **mL** ^**−1**^ **)**	0%	71 ± 2	67 ± 5	68 ± 11	62 ± 5
	aspirin +0%	50 ± 18	29 ± 20	36 ± 28	10 ± 10
	aspirin +25%	26 ± 7	8 ± 4 *	11 ± 5	4 ± 2 *
	aspirin +50%	24 ± 8	7 ± 4 *	7 ± 4 *	3 ± 1 *
	aspirin +100%	15 ± 5	3 ± 2	5 ± 2	2 ± 1
**TRAP‐6 (25 μm)**	0%	75 ± 4	72 ± 4	71 ± 4	67 ± 5
	aspirin +0%	71 ± 4	65 ± 6	69 ± 3	50 ± 13
	aspirin +25%	70 ± 4	61 ± 8	65 ± 5	36 ± 13 *, †
	aspirin +50%	67 ± 6	56 ± 9	62 ± 5	33 ± 15 *
	aspirin +100%	56 ± 5	31 ± 12	46 ± 7	15 ± 8 *, †

Significance is shown as

* *P* < 0.05 *vs*. vehicle,

† *P* < 0.05 *vs*. PGI_2_.

Maximum platelet aggregation to collagen in the presence of PAM‐100% was reduced by the addition of DEA/NONOate (74 ± 4% to 23 ± 9%, *P* < 0.05), PGI_2_ (74 ± 4% to 22 ± 6%, *P* < 0.05) and DEA/NONOate + PGI_2_ (50 ± 10% to 4 ± 1%, *P* < 0.05). Similarly, TRAP‐induced aggregation was reduced in the presence of DEA/NONOate (70 ± 2% to 36 ± 6%, *P* < 0.05), PGI_2_ (67 ± 2% to 35 ± 3%, *P* < 0.05) and DEA/NONOate + PGI_2_ (63 ± 3% to 4 ± 2%, *P* < 0.05). Indeed, even with submaximal PAM‐50% and PAM‐25% P2Y_12_ receptor inhibition, significant inhibition of platelet aggregation was found following addition of DEA/NONOate and PGI_2_.

Although aspirin alone inhibited collagen‐induced platelet aggregation, TRAP‐6‐induced aggregation was only significantly inhibited with the further addition of DEA/NONOate + PGI_2_ to aspirin + PAM‐100% (58 ± 5 to 15 ± 8%, *P* < 0.05), aspirin + PAM‐50% (67 ± 6% to 33 ± 15%, *P* < 0.05) and even aspirin + PAM‐25% (70 ± 4% to 36 ± 13%, *P* < 0.05, Table 2AB).

Collagen (4 μg ml^−1^)‐induced ATP release was significantly inhibited by DEA/NONOate + PGI_2_ in the presence of PAM‐100% (8.2 ± 2.1 nm to 2.3 ± 0.5 nm), PAM‐50% (10.1 ± 2.9 nm to 4.0 ± 1.8 nm, *P* < 0.05) and PAM‐25% (11.0 ± 3.0 nm to 4.1 ± 1.7 nm, *P* < 0.05; Table 2AA), but not by DEA/NONOate or PGI_2_ alone. Similarly, in the presence of aspirin (Table 2AB), collagen‐induced ATP release was significantly reduced by DEA/NONOate + PGI_2_ with submaximal levels of P2Y_12_ blockade (aspirin + PAM‐0%, 8.0 ± 0.5 nm; aspirin + PAM‐25%, 1.9 ± 0.3 nm, *P* < 0.05; aspirin + PAM‐50%, 2.1 ± 0.3, *P* < 0.05; aspirin + PAM‐100%, 1.8 ± 0.2 nm, *P* < 0.05).

**Table 2A:** *In vitro* effects of aspirin and PAM on ATP release. PRP from healthy volunteers (*n* = 4) was treated with PAM (1.5 μm, 3 μm and 6 μm) to represent 25%, 50% and 100% maximum concentration for total P2Y_12_ receptor inhibition, respectively. Tables show ATP release (nm) in response to collagen (4 μg ml^−1^) and TRAP‐6 (25 μm) in the presence of vehicle (NaOH, 10 mm), NO (100 nm), PGI_2_ (1 nm) or NO + PGI_2_ in the (A) absence and (B) presence of aspirin (30 μm).

	**% of maximum concentration for total P2Y**_**12**_ **receptor inhibition**	**Vehicle**	**NO**	**PGI**_**2**_	**NO + PGI**_**2**_
**Collagen (4 μg ml** ^**−1**^ **)**	0%	10.8 ± 3.3	9.8 ± 2.5	7.1 ± 2.4	6.0 ± 1.3
	25%	11.0 ± 3.0	8.8 ± 2.7	7.2 ± 1.6	4.1 ± 1.7
	50%	10.1 ± 2.9	6.4 ± 2.0	8.3 ± 2.9	4.0 ± 1.8
	100%	8.2 ± 2.1	5.2 ± 1.7	6.5 ± 2.2	2.3 ± 0.5 *
**TRAP‐6 (25 μm)**	0%	13.0 ± 1.0	13.0 ± 2.1	11.5 ± 1.5	9.7 ± 1.5
	25%	10.0 ± 2.0	11.7 ± 2.2	12.5 ± 2.5	7.0 ± 2.0
	50%	11.7 ± 2.6	9.3 ± 3.5	13.5 ± 0.5	10.3 ± 1.9
	100%	11.7 ± 3.7	6.0 ± 2.0	10.7 ± 2.9	3.0 ± 1.5

**Table 2B:** 

	**% of maximum concentration for total P2Y**_**12**_ **receptor inhibition**	**Vehicle**	**NO**	**PGI**_**2**_	**NO + PGI**_**2**_
**Collagen (4 μg ml** ^**−1**^ **)**	0%	7.7 ± 1.1	6.6 ± 0.8	7.6 ± 0.6	8.0 ± 0.5
	aspirin +0%	3.4 ± 0.5	3.5 ± 0.2	3.7 ± 0.4	3.3 ± 0.2
	aspirin +25%	3.6 ± 0.2	2.9 ± 0.4	3.0 ± 0.3	1.9 ± 0.3 *
	aspirin +50%	3.4 ± 0.5	2.1 ± 0.3 *	2.3 ± 0.3	2.1 ± 0.3 *
	aspirin +100%	2.9 ± 1.0	1.9 ± 0.5	2.3 ± 0.8	1.8 ± 0.2
**Collagen (10 μg ml** ^**−1**^ **)**	0%	8.8 ± 1.1	8.3 ± 1.1	9.1 ± 0.9	8.4 ± 2.5
	aspirin +0%	5.9 ± 0.9	4.6 ± 0.7	4.8 ± 0.5	3.6 ± 0.5 *
	aspirin +25%	5.6 ± 0.6	4.2 ± 0.2 *	4.5 ± 0.3	3.7 ± 0.4 *
	aspirin +50%	5.2 ± 0.3	4.1 ± 0.1	4.3 ± 0.5	3.5 ± 0.6 *
	aspirin +100%	3.8 ± 0.2	2.4 ± 0.2	3.4 ± 0.4	3.0 ± 0.6
**TRAP‐6 (25 μm)**	0%	11.4 ± 3.0	13.0 ± 1.6	11.7 ± 2.8	10.7 ± 2.9
	aspirin +0%	12.4 ± 0.3	11.6 ± 1.0	10.2 ± 0.7	7.3 ± 1.1 *, †
	aspirin +25%	11.4 ± 1.4	9.1 ± 2.2	10.6 ± 1.3	6.8 ± 1.1
	aspirin +50%	7.9 ± 0.9	7.5 ± 1.2	8.6 ± 1.6	5.3 ± 1.2
	aspirin +100%	10.7 ± 2.1	8.7 ± 1.8	9.5 ± 1.6	5.0 ± 1.1 *

Significance is shown as

* *P* < 0.05 *vs*. vehicle,

† *P* < 0.05 *vs.* PGI_2_.

### Synergy between PGI_2_, DEA/NONOate and P2Y_12_ blockade

**Figure 2:**
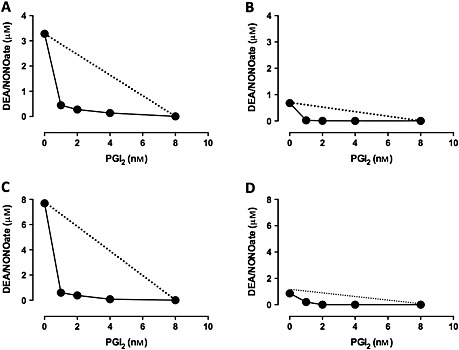
Synergism between NO, PGI_2_ and PAM. I*C*_50_ isobolograms were generated by analyzing combinations of NO and PGI_2_ required to produce a 50% inhibition of platelet aggregation stimulated by collagen (30 μg ml^−1^) in the (A) absence and (B) presence of PAM (6 μm) and by TRAP‐6 (25 μm) in the (C) absence and (D) presence of PAM. The linear relationship is predicted by the arithmetic sum of the effect of either NO or PGI_2_ alone, as described in the methods, and the experimental line curving towards the axes indicates a strong, synergistic relationship. *n* = 4 for each point

Isobolographic analyses indicated strong synergistic inhibition between DEA/NONOate and PGI_2_ against platelet aggregation induced by collagen (30 μg ml^−1^, Figure 2A) or TRAP‐6 amide (25 μm, Figure 2B), with isoboles curving strongly towards the axes. P2Y_12_ blockade caused a further powerful (5‐fold and 10‐fold, respectively) enhancement in the synergy between DEA/NONOate and PGI_2_ for the inhibition of aggregations induced by collagen (Figure 2B) and TRAP‐6 (Figure 2D).

### Involvement of cAMP and cGMP in the synergistic effects of P2Y_12_ blockade, PGI_2_ and DEA/NONOate

**Figure 3:**
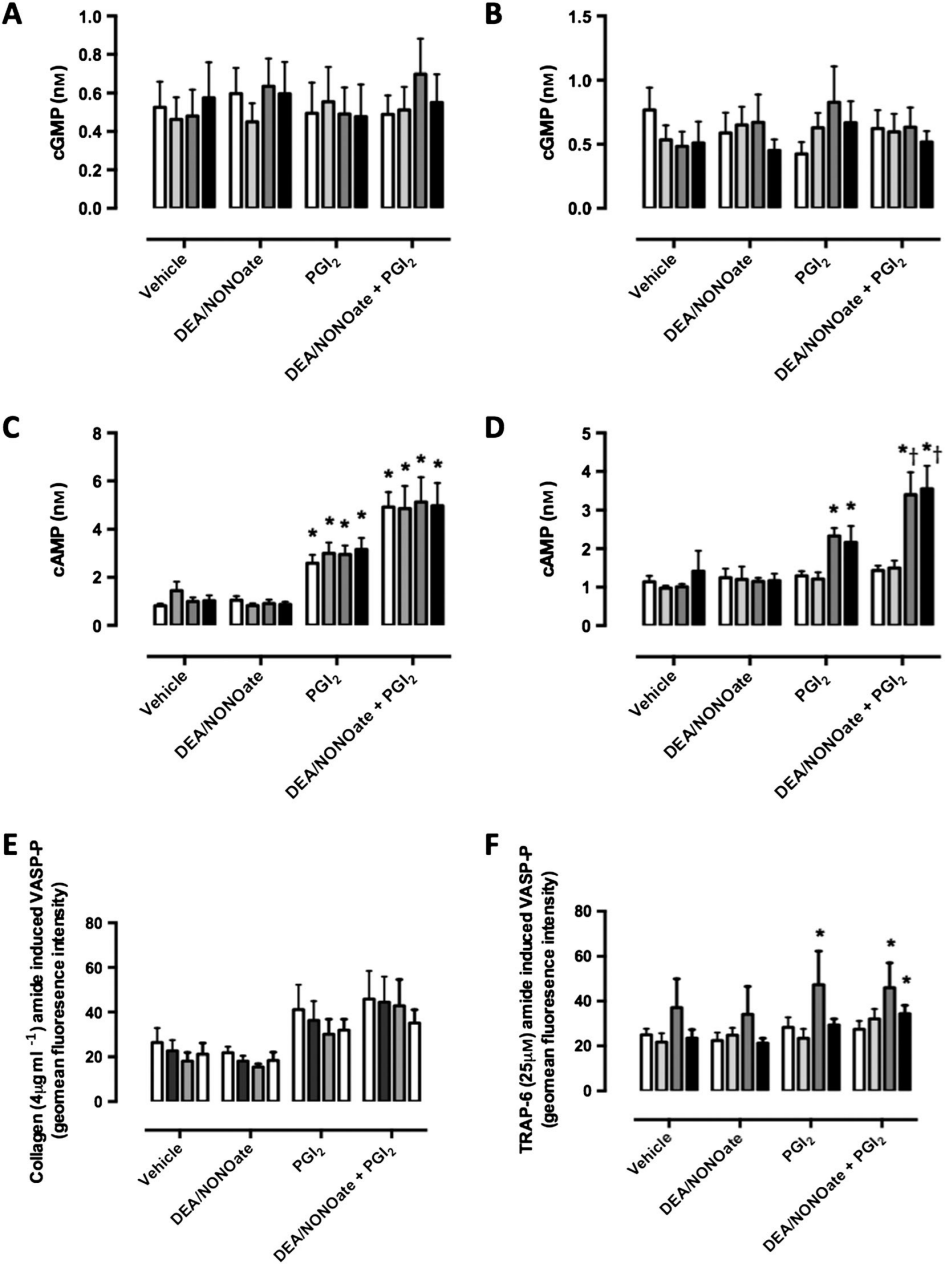
*In vitro* effects of aspirin and PAM on cyclic nucleotide levels. PRP from healthy volunteers (*n* = 4) was treated with aspirin (30 μm), PAM (6 μm) or both followed by addition of vehicle (NaOH, 10 mm), NO (100 nm), PGI_2_ (1 nm) or NO + PGI_2_. cGMP levels were then measured following stimulation by (A) collagen (4 μg ml^−1^) and (B) TRAP‐6 (25 μm) as were cAMP levels following (C) collagen and (D) TRAP‐6. Phospho (Ser^239^)‐VASP levels in (E) collagen and (F) TRAP‐6‐stimulated PRP were determined by flow cytometry as a composite of cGMP and cAMP responses. Significance is shown as * *P* < 0.05 *vs.* non‐treated and † *P* < 0.05 PGI_2_ with corresponding PAM or aspirin + PAM. (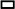) vehicle, (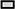) aspirin, (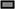) pam, (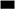) aspirin + PAM

We found no significant change in cGMP levels in the platelets in response to DEA/NONOate and/or PGI_2_ after incubation with aspirin, PAM or aspirin + PAM after platelet aggregation stimulated by collagen (Figure 3A) or TRAP‐6 (Figure 3B).

In collagen‐stimulated platelets, cAMP levels (0.8 ± 0.1 nm) were not altered by DEA/NONOate, but were significantly increased by PGI_2_ (2.6 ± 0.3 nm, *P* < 0.05) and even more so by the combination of DEA/NONOate + PGI_2_ (4.9 ± 0.6 nm, *P* < 0.05). Neither PAM, aspirin nor PAM + aspirin altered the cAMP response in collagen‐stimulated platelets (Figure 3C). In contrast, in TRAP‐6‐stimulated platelets, DEA/NONOate, PGI_2_ and the combination did not elevate cAMP levels in vehicle or aspirin groups, but did in the presence of PAM (1.0 ± 0.1 nm to 2.3 ± 0.2 nm, *P* < 0.05) or PAM + aspirin (1.4 ± 0.5 nm to 2.2 ± 0.4 nm, *P* < 0.05), PGI_2_ increased cAMP levels and this response was further enhanced by addition of DEA/NONOate (PAM 3.4 ± 0.6 nm, PAM + aspirin 3.5 ± 0.6 nm, *P* < 0.05, Figure 3D).

Phospho (Ser^239^)‐VASP, a downstream marker of PKG activation, remained unchanged in most conditions studied but was increased following TRAP‐6 stimulation in the presence of PAM in all cases and most so in the presence of PGI_2_ (28 ± 5 to 47 ± 15 units, *P* < 0.05) or DEA/NONOate + PGI_2_ (27 ± 4 to 46 ± 11 units, *P* < 0.05, Figure 3F).

## Discussion

Here we show in healthy individuals receiving standard DAPT leading to consensus levels of platelet inhibition that *ex vivo* responses to the strong primary platelet activators collagen and TRAP‐6 are powerfully influenced by the presence of NO and PGI_2_. The strong synergies between P2Y_12_ inhibitors and the cAMP and cGMP signalling systems mean that the *in vivo* platelet reactivity in patients receiving DAPT will be a function of the level of P2Y_12_ receptor blockade and the levels of endothelium‐derived NO and PGI_2_. This provides an explanation for different thrombotic outcomes in the presence of similar levels of platelet blockade, i.e. individual patients with different levels of endothelial function, or indeed disease‐driven endothelial dysfunction, would have different levels of *in vivo* platelet inhibition for the same level of DAPT activity, as determined by *ex vivo* testing.

DAPT, aspirin plus a P2Y_12_ receptor blocker, is the preventative therapy provided to patients at particular risk of coronary thrombosis, notably for the first 12 months following coronary stent implantation or an acute coronary syndrome 20, 21. Despite this therapeutic approach coronary thrombosis still occurs, and there have been great efforts made to find *ex vivo* tests that could predict for clinical outcomes 22, 23. Deductive reasoning leads to the conclusion that less effective platelet blockade would leave individuals at increased risk of thrombosis and so multiple efforts have been made to link levels of platelet reactivity in *ex vivo* tests to clinical outcomes. Despite the attractive logic of this approach, tailoring anti‐platelet therapy to *ex vivo* platelet responses has failed to provide any improvement in clinical outcomes as noted in large scale studies such as ADRIE 24 and several large scale prospective, randomized clinical trials, such as GRAVITAS 7, ARCTIC 8, TRIGGER‐PCI 9 and TRILOGY 10.

In patients receiving clopidogrel, there are well‐characterized metabolic differences that can produce suboptimal levels of its active metabolite and consequently result in suboptimal levels of P2Y_12_ receptor blockade 25. There are also some reports of variability in the effects of prasgurel and ticagrelor, although to a much lesser extent than for clopidogrel 26. Biochemical resistance to the effects of aspirin are also particularly rare 27. Allowing for differences dependent upon adherence to therapy, individuals on DAPT may in fact present a rather more homogenous level of platelet inhibition than can be associated to different clinical outcomes. Having recently reported that blockade of P2Y_12_ receptors greatly increases the inhibitory effects of NO, and knowing that not only was there a similar interaction with the inhibitory effects of PGI_2_ but that NO and PGI_2_ powerfully synergize to inhibit platelets, we reasoned that differences in the levels of NO and PGI_2_ in the presence of the same levels of P2Y_12_ receptor blockade would produce different levels of platelet inhibition. By testing this hypothesis in individuals receiving standard DAPT we show here that strong and synergistic interactions between P2Y_12_ receptor blockade and endothelium‐derived mediators produce profound inhibitory effects upon platelets. We firstly established that the drug regime given in our studies elicited satisfactory reduction in baseline reactivity, therefore establishing effectiveness of P2Y_12_ and/or COX inhibition in accordance with suggested analytical cutoffs 28. These reductions were against high pre‐treatment levels of platelet reactivity (>70% response to 5 μm ADP) 29. In these studies, and others presented here, we took care to include the standard measures of platelet function as determined in consensus statements 28, 30. Then to make data readily accessible we have presented results in the form of heat maps that move from red to green, indicating movement from full platelet activation to no platelet activation.

It is well known that NO and PGI_2_ synergize to inhibit platelets 12 and it has been demonstrated by ourselves and others that P2Y_12_ antagonists potentiate the inhibitory actions of both PGI_2_, dependent upon cAMP 17, and NO, dependent upon cGMP generation 18. Here, we have shown that this synergy, in the presence of NO and PGI_2_ is mostly cAMP dependent. Therefore phosphodiesterase 3 (PDE3) inhibitors, such as cilostazol, may have an enhanced effect compared with PDE5 inhibitors, such as sildenafil. Though not widely commented upon, the body contains many more endothelial cells than platelets, in the order of 50 times more, and the two populations constantly interact. In the circulation DAPT exerts its effects upon platelets in the presence of endothelium‐derived mediators while these are absent in *ex vivo* testing. In the studies presented here we found that the interactions of NO, PGI_2_ and P2Y_12_ receptor blockade in inhibiting platelets were markedly synergistic as noted by isobolographic analysis and measures of aggregation, ATP release, activation of GP IIb/IIIa receptors and P‐selectin expression. In volunteers taking DAPT we noted inhibition of responses to ADP and AA that were in keeping with consensus statements of effective DAPT, i.e. in our study the drugs were working to an effective level of clinical efficacy. Despite this level of effective inhibition, high concentrations of the strong primary platelet activators, TRAP‐6 or collagen, still caused notable platelet activation. Addition of low concentrations of NO and PGI_2_, to model the environment within the blood vessel, had little effect on their own but led to almost complete inhibition in platelets from individuals treated with DAPT. Similarly, while NO, PGI_2_ or DAPT alone had relatively little effect upon platelet granule release, determined as ATP release, when combined they caused more than 50% inhibition. These results indicate that even in the presence of effective DAPT, i.e. within consensus guidelines, the presence of NO and PGI_2_ lead to very much higher levels of platelet inhibition.

Next using an *in vitro* approach we modelled events in the presence of suboptimal levels of P2Y_12_ receptor blockade by using concentrations of PAM that were 50% and 25% of the effective concentration. Under these conditions we noted that relative to the consensus levels of DAPT we did not achieve significant reduction in platelet aggregation. Notably, however, in the presence of NO and PGI_2_ effective levels of inhibition were achieved, even when platelets were exposed to only 25% of the effective concentration of PAM. As we express in heat maps, there is a clear interaction between DAPT and the endothelial mediators that move platelets from reactive (‘red’) to unreactive (‘green’). Interestingly, these comparisons indicate that 25% of the effective concentration of PAM plus NO and PGI_2_ produces a stronger inhibition in LTA, the ‘gold standard test’, than 100% of the effective concentration of PAM in the absence of NO and PGI_2_ (i.e. the normal conditions for testing *ex vivo* platelet responsiveness). This suggests that in individuals in whom suboptimal P2Y_12_ inhibition is achieved, such as poor clopidogrel metabolizers, anti‐platelet efficacy may be particularly sensitive to any changes in endothelial function. Our *in vitro* data also demonstrate that the triple synergy between P2Y_12_ blockade, NO and PGI_2_ can be explained by changes in cAMP signalling, which is consistent with known interactions between NO and PGI_2_ 31 and PGI_2_ and P2Y_12_ 17.

**Figure 4:**
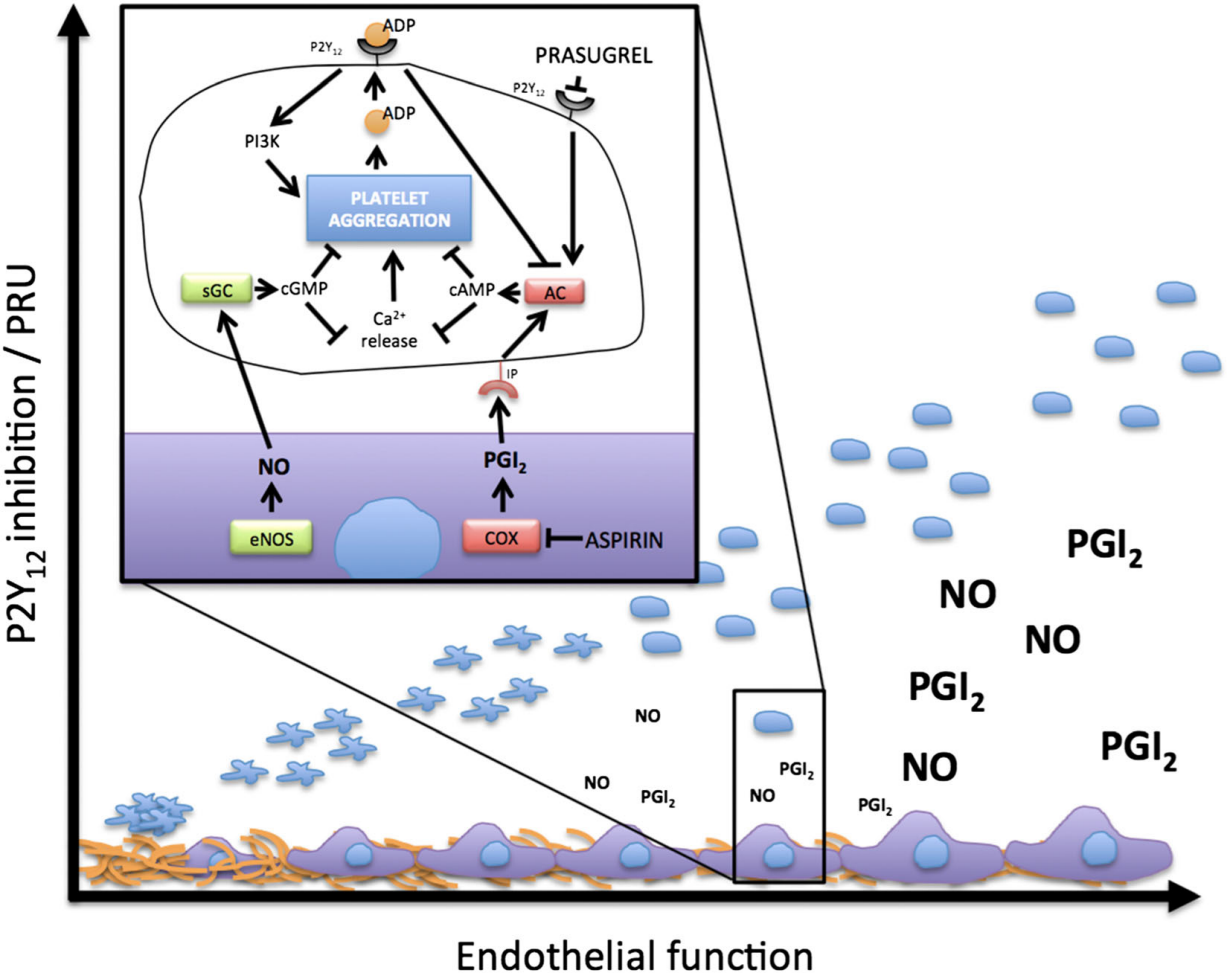
Summary of the interaction between the endothelium and P2Y_12_ antagonism. In the healthy intact circulation platelets are kept in a non‐activated state in part by the action of endothelium‐derived mediators, NO and PGI_2_. At areas of endothelial damage platelets become activated leading to platelet adherence and activation. This effect is partly driven by stimulation of P2Y_12_ receptors following from platelet release of ADP. This stimulation of P2Y_12_ receptors inhibits the effects of cAMP and cGMP within platelets, making platelets more excitable, and amplifying platelet activation. When P2Y_12_ receptors are blocked, cAMP and cGMP pathways are not inhibited by ADP and the inhibitory effects of NO and PGI_2_ are sustained. The inhibitory effectiveness of P2Y_12_ receptor blockade and DAPT *in vivo* is therefore strongly dependent upon the production of NO and PGI_2_ within the circulation

We show that following standard DAPT the level of platelet reactivity is a function of the level of P2Y_12_ receptor blockade and the levels of NO and PGI_2_. While we added NO and PGI_2_ exogenously they are surrogates for the effects of endogenous NO and PGI_2_ and other elevators of platelet cyclic nucleotides such as adenosine. We propose that, since *in vivo* platelet function is a product of both internal platelet responsive signalling reactivity and the external influence of the endothelium, an assessment of endothelial mediator production could be combined with results from *ex vivo* platelet testing to predict thrombotic outcomes better in individual patients. Furthermore, with the emergence of this complex and very powerful synergy between PGI_2_, NO and P2Y_12_ inhibitors (Figure 4), we should perhaps consider optimizing the availability and activity of endothelium‐derived mediators (such as PDE inhibitors), or providing mimetic drugs, rather than adding in further anti‐platelet therapies. These findings could, eventually, be applied in a personalized medicine framework where the endothelial mediator production of individuals is assessed and appropriate add‐on therapy applied. In a more generalized approach these additional therapies could also be supplied to patient groups with known endothelial dysfunction, such as diabetics. This approach could provide increased anti‐platelet efficacy while avoiding the increased risk of bleeding events associated with the approach of triple anti‐platelet therapy.

## Competing Interests

All authors have completed the Unified Competing Interest form at http://www.icmje.org/coi_disclosure.pdf (available on request from the corresponding author) and declare RBMK, MVC, MHL and PCJA had support from the British Heart Foundation [FS/12/53/29 643 to RBMK, PG/11/75/29 105 to MVC and MHL, PG/12/68/29 779 to PCJA] and all authors had support from the William Harvey Research Foundation for the submitted work. TDW has received research grants from AstraZeneca relating to clinical development of P2Y_12_ inhibitors in the previous 3 years. There are no other relationships or activities that could appear to have influenced the submitted work.

## Contributors

RBMK, ATT, NSK and TDW designed the study and experiments. MVC, RBMK, MHL, NAM, NSK and PCJA collected data. MVC, NAM and RBMK performed data analysis. MVC, RBMK and TDW drafted the manuscript. All authors contributed to the writing of the manuscript. MVC and RBMK contributed equally to this work as first authors.

*We thank Ivana Vojnovic and Chih‐Chin Shih for technical assistance*.

## Supporting information


**Figure S1** Standard platelet aggregation tests. Standard light transmission aggregometry responses to AA (1 mm), ADP (5 μm), collagen (0.4 μg ml^−1^) and U46619 (10 μm) in healthy volunteers before and following treatment with (A) aspirin (75 mg), (B) prasugrel (10 mg) or (C) DAPT (aspirin, 75 mg, plus prasugrel, 10 mg) for 7 days. *n* = 8 for all. Significance is shown as * *P* < 0.05 *vs*. non‐treated
**Figure S2** The effect of DAPT on platelet aggregation and ATP release. Representative light transmission aggregometry traces of PRP before and after DAPT (aspirin, 75 mg, plus prasugrel, 10 mg) treatment in the presence of vehicle (NaOH, 10 mm), DEA/NONOate (100 nm), PGI_2_ (1 nm) or DEA/NONOate + PGI_2_ following stimulation with (A) collagen (4 μg ml^−1^) or (B) TRAP‐6 amide (25 μm). (C) Representative lumi‐aggregometry traces in the same conditions after TRAP‐6 amide (25 μm) stimulation, where ATP release is measured as an increase in voltage
**Figure S3** The effect of aspirin and prasugrel on platelet aggregation. Healthy volunteers (*n* = 8) were treated with aspirin (75 mg) or prasugrel (10 mg) for 7 days. Aggregometry was conducted in the presence of vehicle (NaOH, 10 mm), DEA/NONOate (100 nm), PGI_2_ (1 nm) or DEA/NONOate + PGI_2_ before and after aspirin, using as agonists (A) collagen (4 μg ml^−1^) or (B) TRAP‐6 amide (25 μm), and before and after prasugrel, also using (C) collagen (4 μg ml^−1^) or (D) TRAP‐6 amide (25 μm). Data are presented as final aggregation (%, mean ± SEM). Summary heatmaps after stimulation with (E) collagen (4 μg ml^−1^) or (F) TRAP‐6 amide (25 μm) indicate maximum aggregation with red and minimum aggregation with green, before treatment and after aspirin (75 mg), prasugrel (10 mg) or DAPT for 7 days. *n* = 8 for all. Significance is shown as * *P* < 0.05 *vs*. non‐treated, † *P* < 0.05 *vs*. NaOH drug‐treated ‡ *P* < 0.05 *vs.* PGI_2_ drug‐treated
**Figure S4** The effect of aspirin and prasugrel on platelet ATP release. Healthy volunteers (*n* = 8) were treated with aspirin (75 mg) or prasugrel (10 mg) for 7 days. Lumi‐aggregometry was conducted in the presence of vehicle (NaOH, 10 mm), DEA/NONOate (100 nm), PGI_2_ (1 nm), or DEA/NONOate + PGI_2_ before and after (A) aspirin or (B) prasugrel, using collagen (4 μg ml^−1^) as an agonist. Data are presented as maximum ATP release (%, mean ± SEM). (C) A summary heatmap after stimulation with collagen (4 μg ml^−1^) indicates maximum ATP release with red and minimum ATP release with green before treatment and after aspirin (75 mg), prasugrel (10 mg) or DAPT for 7 days. *n* = 8 for all. Significance is shown as * *P* < 0.05 *vs*. non‐treated, † *P* < 0.05 *vs*. NaOH drug‐treated ‡ *P* < 0.05 *vs*. PGI_2_ drug‐treated
**Figure S5** Representative control data for flow cytometry experiments. GPIIb/IIIa activation by PAC‐1 binding in the (A) absence and (B) presence of DAPT, P‐selectin expression in the (C) absence and (D) presence of DAPT and VASP phosphorylation (Ser_239_) in the (E) absence and (F) presence of DAPT was measured by flow cytometry in PRP stimulated with TRAP‐6 (25 μm) in the presence of vehicle (NaOH, 10 mm), DEA/NONOate (100 nm), PGI_2_ (1 nm) or DEA/NONOate + PGI_2_. Histograms are representative of *n* = 3
**Figure S6** The effect of aspirin, prasugrel and DAPT on P‐selectin and glycoprotein IIb/IIIa. Healthy volunteers (*n* = 8) were treated with aspirin (75 mg), prasugrel (10 mg), or DAPT for 7 days. Heatmaps in response to TRAP‐6 amide (25 μm)‐stimulated PRP before and after DAPT treatment in the presence of vehicle (NaOH, 10 mm), DEA/NONOate (100 nm), PGI_2_ (1 nm) or DEA/NONOate + PGI_2_ were generated for (A) CD62P (P‐selectin) and (B) PAC‐1 (GPIIb/IIIa binding). Red represents maximum expression and green shows minimum expression with each cell representing data from three groups of eight subjects

Supporting info item

Supporting info item

Supporting info item

Supporting info item

Supporting info item

Supporting info item

Supporting info item
