# Supplementary material for: P2Y12 receptor blockade synergizes strongly with nitric oxide and prostacyclin to inhibit platelet activation
Source: Br J Clin Pharmacol. 2016 Feb 10;81(4):621–33. doi: 10.1111/bcp.12826 (PMC4799935; doi:10.1111/bcp.12826)
Supplement: Supplementary file 1 — Supporting info item [file BCP-81-621-s001.doc]

**SUPPORTING MATERIALS**

**P2Y_12_ Receptor Blockade Synergises Strongly with Nitric Oxide and Prostacyclin to Inhibit Platelet Activation**

Melissa V. Chan, PhD^1*;^ Rebecca B.M. Knowles, MD^1*^; Martina H. Lundberg, PhD^1^; Arthur T. Tucker, PhD^1^; Nura A Mohamed, MRes^2,3;^ Nicholas S. Kirkby, PhD^1,3^; Paul C.J. Armstrong, PhD^1^; Jane A Mitchell, PhD^3^; Timothy D. Warner, PhD^1^

**^1^** The William Harvey Research Institute, Barts and the London School of Medicine and Dentistry, Queen Mary University of London, London, United Kingdom.

^2^ Qatar Foundation Research and Development Division, Doha, Qatar

^3^ National Heart & Lung Institute, Imperial College London, London, United Kingdom

*These authors contributed equally to the manuscript and their names appear in alphabetical order

**Address for Correspondence:**

Timothy D. Warner, PhD

The William Harvey Research Institute

Barts and the London School of Medicine and Dentistry

Charterhouse Square

London

EC1M 6BQ, UK

Telephone: + 44 20 7882 2100

Fax: + 44 20 7882 8252

Email: t.d.warner@qmul.ac.uk

**SUPPORTING LEGENDS TO FIGURES**

**Supplementary Figure 1. Standard platelet aggregation tests.** Standard light transmission aggregometry responses to AA (1 mM), ADP (5 µM), collagen (0.4 µg/mL) and U46619 (10 µM) in healthy volunteers before and following treatment with (A) aspirin (75 mg), (B) prasugrel (10 mg), or (C) DAPT (aspirin, 75 mg, plus prasugrel, 10 mg) for 7 days. N=8 for all. Significance is shown as * p<0.05 vs non-treated.

**Supplementary Figure 2. The effect of DAPT on platelet aggregation and ATP release.** Representative light transmission aggregometry traces of PRP before and after DAPT (aspirin, 75 mg, plus prasugrel, 10 mg) treatment in the presence of vehicle (NaOH, 10 mM), DEA/NONOate (100 nM), PGI_2_ (1 nM), or DEA/NONOate+PGI_2_ following stimulation with (A) collagen (4 μg/mL) or (B) TRAP-6 amide (25 μM). (C) Representative lumi-aggregometry traces in the same conditions after TRAP-6 amide (25 μM) stimulation, where ATP release is measured as an increase in voltage.

**Supplementary Figure 3.** **The effect of aspirin and prasugrel on platelet aggregation.** Healthy volunteers (n = 8) were treated with aspirin (75 mg) or prasugrel (10 mg) for 7 days. Aggregometry was conducted in the presence of vehicle (NaOH, 10 mM), DEA/NONOate (100 nM), PGI_2_ (1 nM), or DEA/NONOate + PGI_2_ before and after aspirin, using as agonists (A) collagen (4 µg/mL) or (B) TRAP-6 amide (25 µM), and before and after prasugrel, also using (C) collagen (4 µg/mL) or (D) TRAP-6 amide (25 µM). Data are presented as final aggregation (%, mean±SEM). Summary heatmaps after stimulation with (E) collagen (4 µg/mL) or (F) TRAP-6 amide (25 µM) indicate maximum aggregation with red and minimum aggregation with green, before treatment and after aspirin (75 mg), prasugrel (10 mg), or DAPT for 7 days. N = 8 for all. Significance is shown as * p<0.05 vs non-treated, † p<0.05 vs NaOH drug-treated ‡ p<0.05 vs PGI­_2_ drug-treated.

**Supplementary Figure 4.** **The effect of aspirin and prasugrel on platelet ATP release.** Healthy volunteers (n = 8) were treated with aspirin (75 mg) or prasugrel (10 mg) for 7 days. Lumi-aggregometry was conducted in the presence of vehicle (NaOH, 10 mM), DEA/NONOate (100 nM), PGI_2_ (1 nM), or DEA/NONOate + PGI_2_ before and after (A) aspirin or (B) prasugrel, using collagen (4 µg/mL) as an agonist. Data are presented as maximum ATP release (%, mean±SEM). (C) A summary heatmap after stimulation with collagen (4 µg/mL) indicates maximum ATP release with red and minimum ATP release with green before treatment and after aspirin (75 mg), prasugrel (10 mg) or DAPT for 7 days. N = 8 for all. Significance is shown as * p<0.05 vs non-treated, † p<0.05 vs NaOH drug-treated ‡ p<0.05 vs PGI­_2_ drug-treated.

**Supplementary Figure 5**. **Representative control data for flow cytometry experiments.** GPIIb/IIIa activation by PAC-1 binding in the **(**A**)** absence and **(**B**)** presence of DAPT, P-selectin expression in the **(**C**)** absence and **(**D**)** presence of DAPT and VASP phosphorylation (Ser_239_) in the **(**E**)** absence and **(**F**)** presence of DAPT was measured by flow cytometry in PRP stimulated with TRAP-6 (25 μM) in the presence of vehicle (NaOH, 10 mM), DEA/NONOate (100 nM), PGI_2_ (1 nM), or DEA/NONOate + PGI_2_. Histograms are representative of n = 3.

**Supplementary Figure 6. The effect of aspirin, prasugrel and DAPT on P-selectin and glycoprotein IIb/IIIa.** Healthy volunteers (n = 8) were treated with aspirin (75 mg), prasugrel (10 mg), or DAPT for 7 days. Heatmaps in response to TRAP-6 amide (25 μM)-stimulated PRP before and after DAPT treatment in the presence of vehicle (NaOH, 10 mM), DEA/NONOate (100 nM), PGI_2_ (1 nM) or DEA/NONOate+PGI_2_ were generated for (A) CD62P (P-selectin) and (B) PAC-1 (GPIIb/IIIa binding). Red represents maximum expression and green shows minimum expression with each cell representing data from 3 groups of 8 subjects.
