# Supplementary figures and images for: P2Y12 receptor blockade synergizes strongly with nitric oxide and prostacyclin to inhibit platelet activation
Source: Br J Clin Pharmacol. 2016 Feb 10;81(4):621–33. doi: 10.1111/bcp.12826 (PMC4799935; doi:10.1111/bcp.12826)

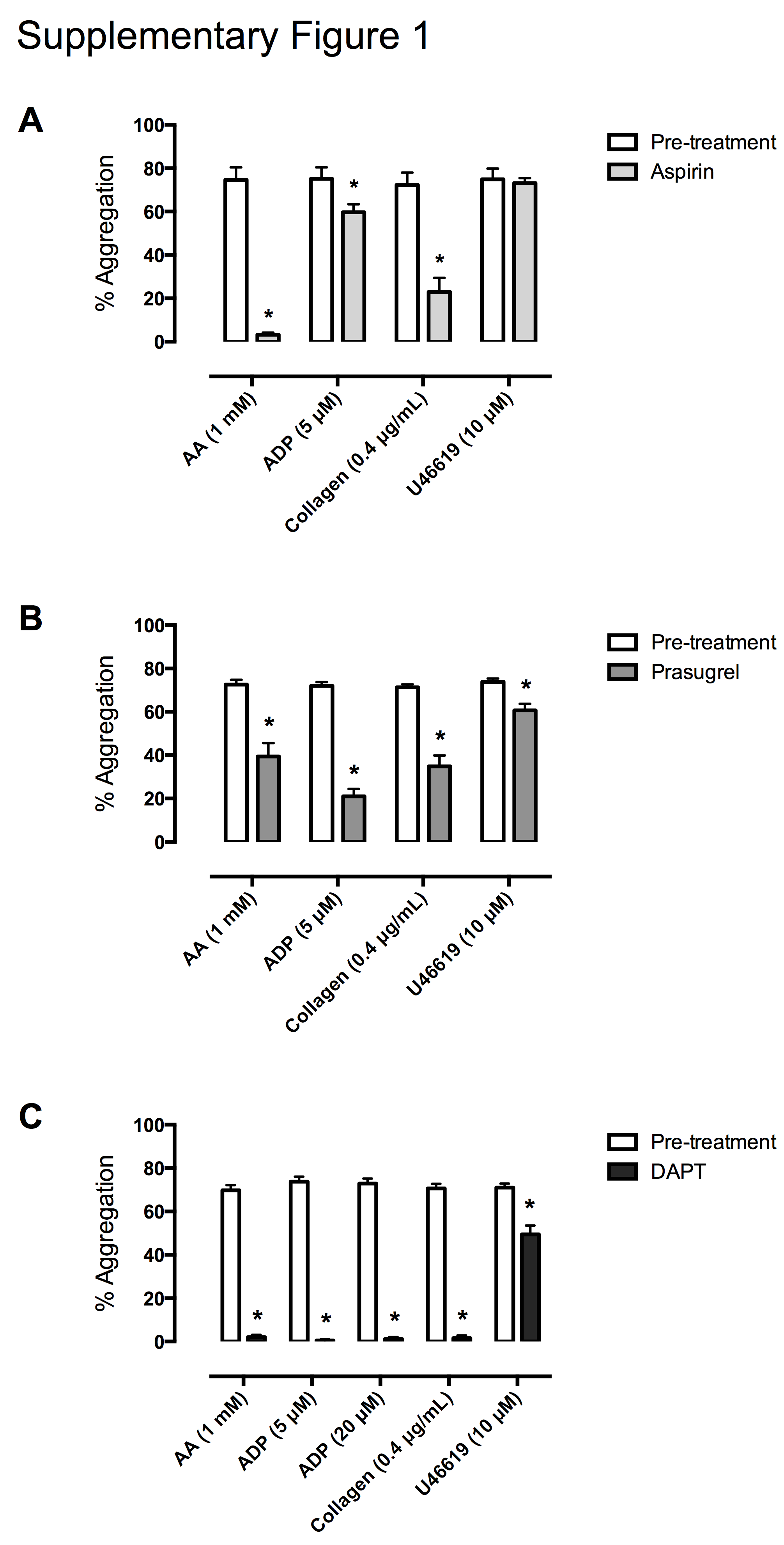

Supplement: Supplementary file 2 — Supporting info item [file BCP-81-621-s002.tiff]

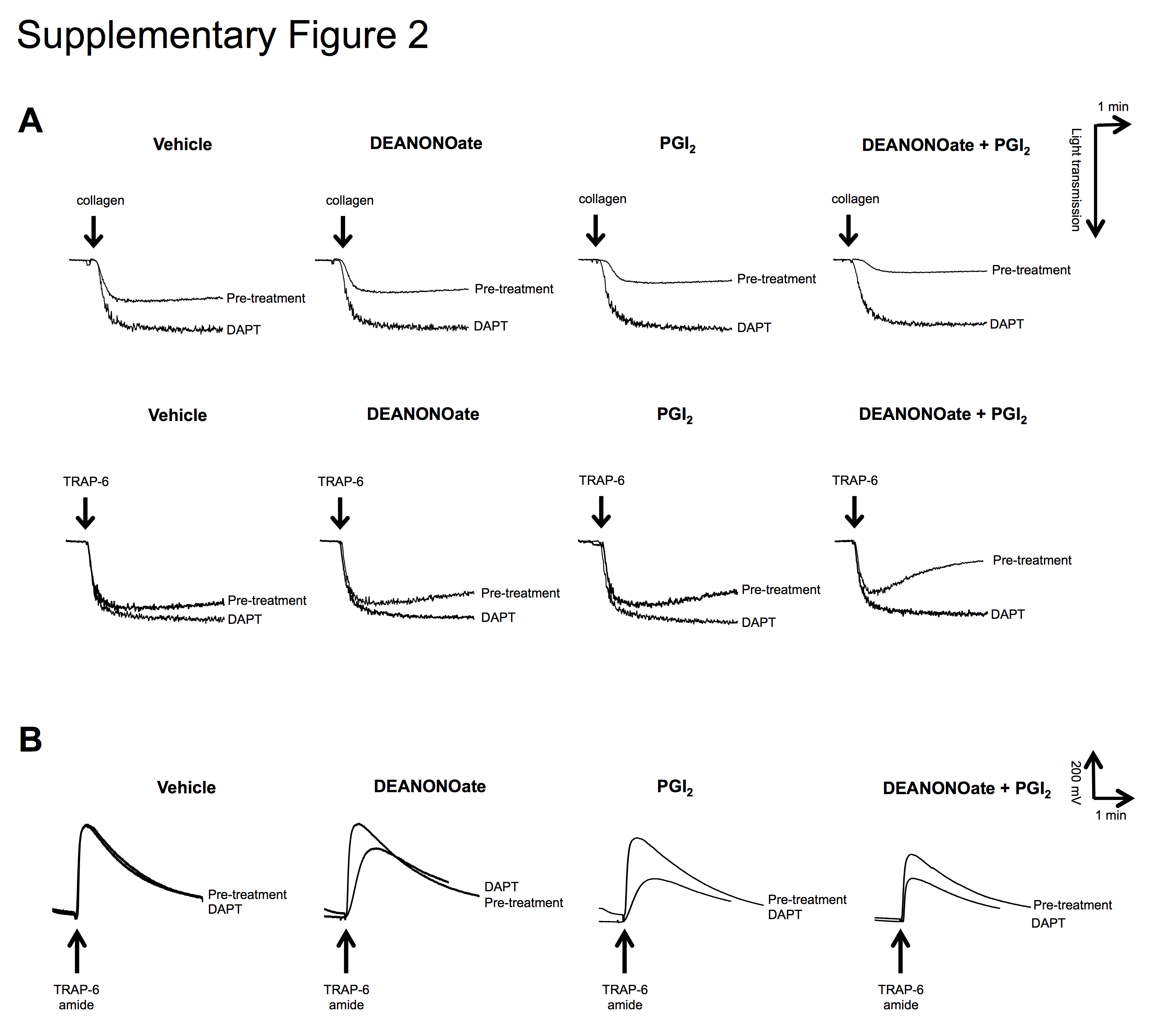

Supplement: Supplementary file 3 — Supporting info item [file BCP-81-621-s003.tiff]

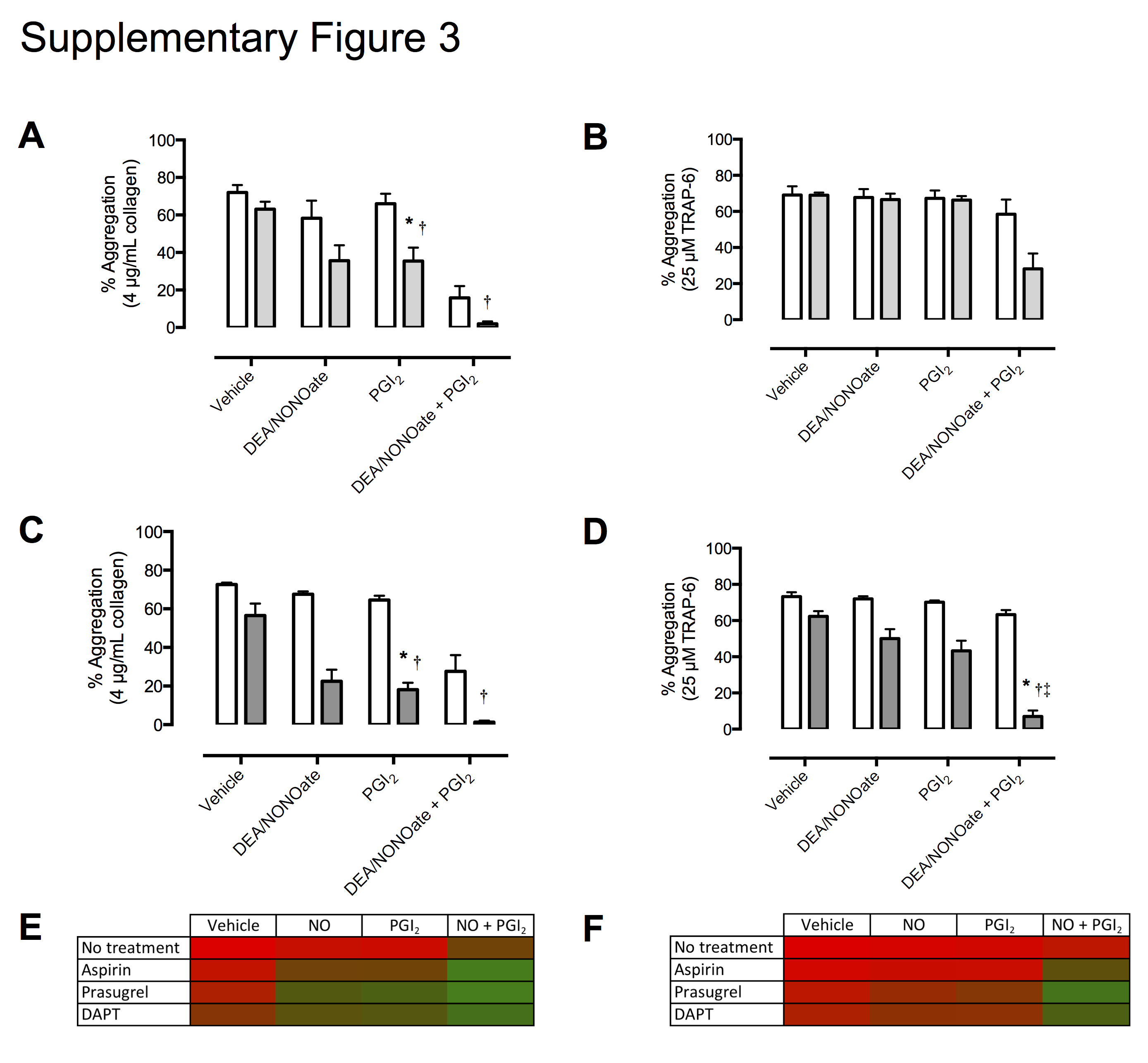

Supplement: Supplementary file 4 — Supporting info item [file BCP-81-621-s004.tiff]

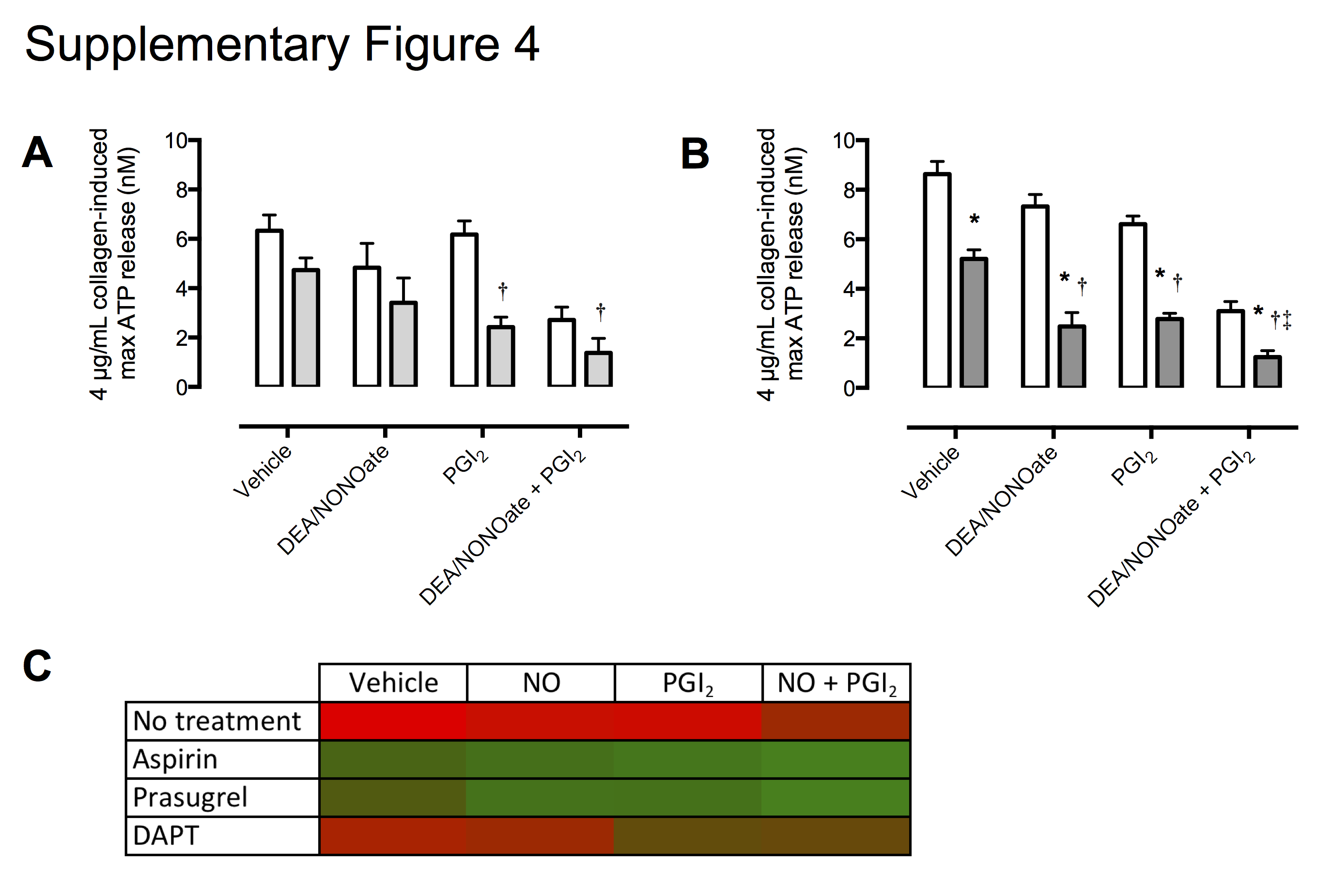

Supplement: Supplementary file 5 — Supporting info item [file BCP-81-621-s005.tiff]

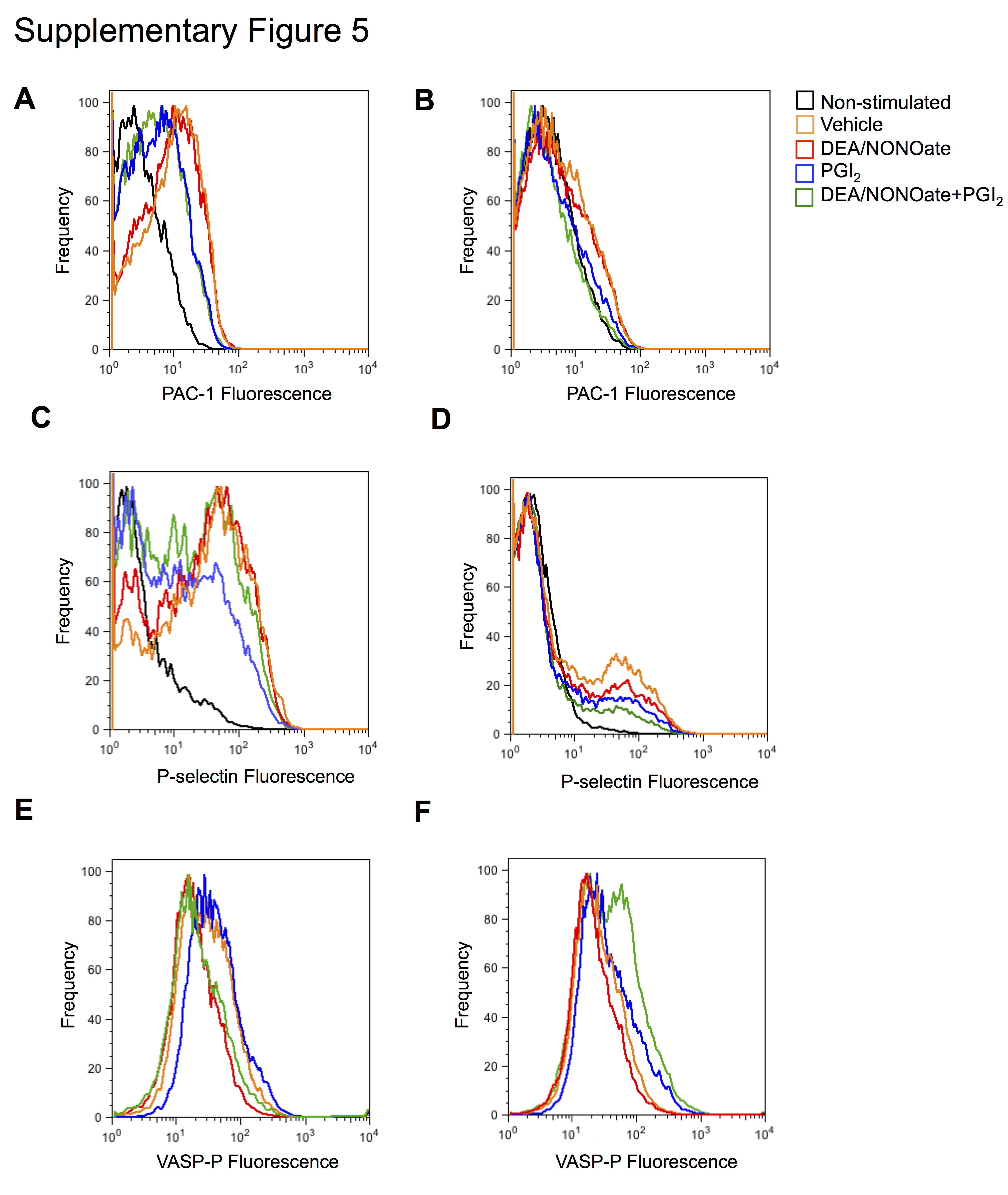

Supplement: Supplementary file 6 — Supporting info item [file BCP-81-621-s006.tiff]

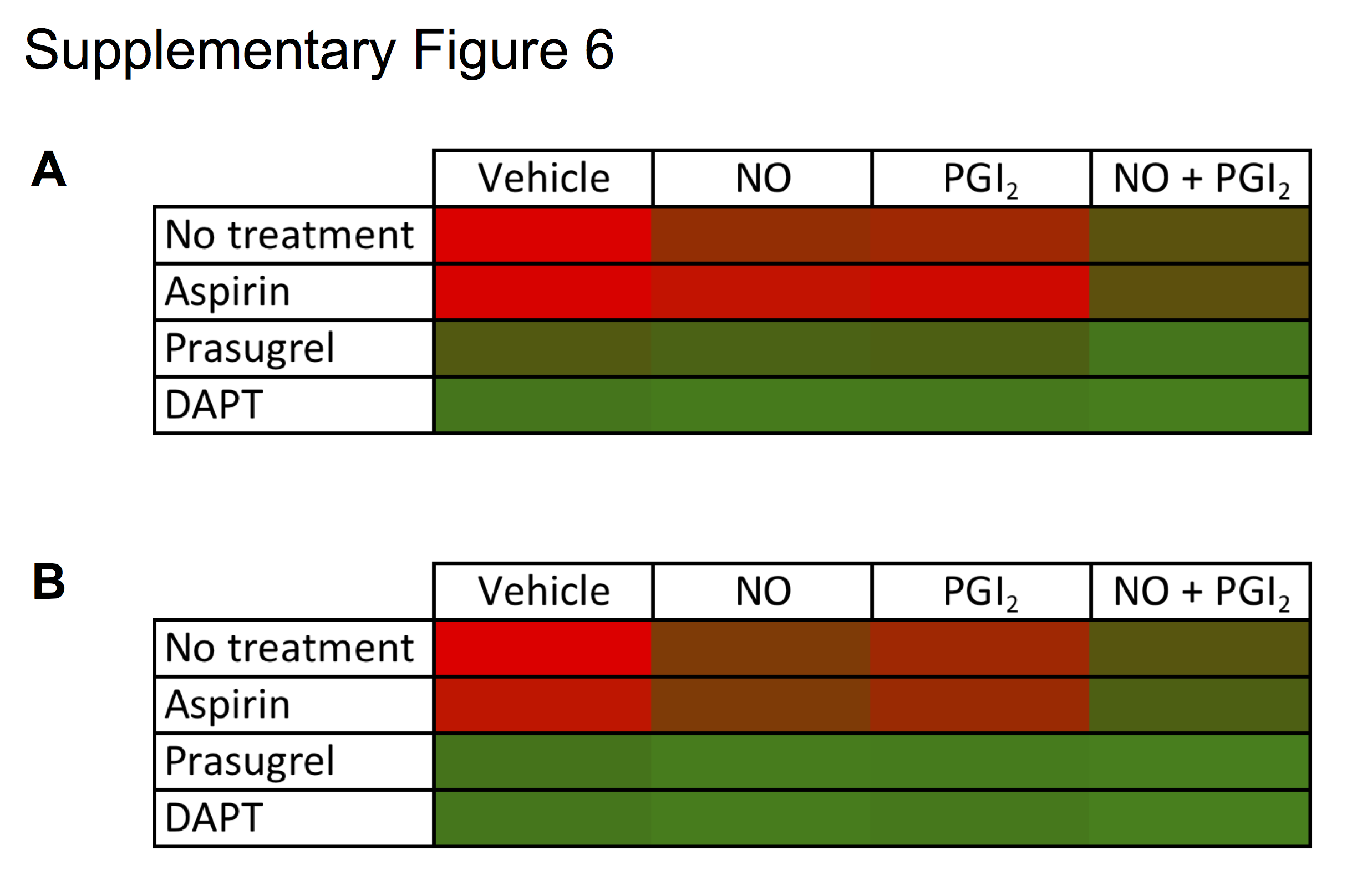

Supplement: Supplementary file 7 — Supporting info item [file BCP-81-621-s007.tiff]
